# Predictive modelling and identification of critical variables of mortality risk in COVID-19 patients

**DOI:** 10.1038/s41598-023-46712-w

**Published:** 2025-01-16

**Authors:** Olawande Daramola, Tatenda Duncan Kavu, Maritha J. Kotze, Jeanine L. Marnewick, Oluwafemi A. Sarumi, Boniface Kabaso, Thomas Moser, Karl Stroetmann, Isaac Fwemba, Fisayo Daramola, Martha Nyirenda, Susan J. van Rensburg, Peter S. Nyasulu

**Affiliations:** 1 Department of Information Technology, Cape Peninsula University of Technology, Cape Town, South Africa; 2 Division of Epidemiology and Biostatistics, Faculty of Medicine, and Health Sciences, Stellenbosch University, Cape Town, South Africa; 3 Division of Chemical Pathology, Department of Pathology, Faculty of Medicine and Health Sciences, Stellenbosch University, Cape Town, South Africa; 4 Division of Chemical Pathology, Department of Pathology, Faculty of Medicine and Health Sciences, Stellenbosch University, Cape Town, South Africa; 5 Applied Microbial and Health Biotechnology Institute, Cape Peninsula University of Technology, Cape Town, South Africa; 6 St Pölten University of Applied Sciences, St Pölten, Austria; 7 School of Health Information Science, University of Victoria, Victoria, BC Canada; 8 Department of Mathematics and Computer Science, Philipps University of Marburg, Hans-Meerwein Str. 6 D-35032, Marburg, Germany

**Keywords:** Diseases, Health care, Mathematics and computing

## Abstract

South Africa was the most affected country in Africa by the coronavirus disease 2019 (COVID-19) pandemic, where over 4 million confirmed cases of COVID-19 and over 102,000 deaths have been recorded since 2019. Aside from clinical methods, artificial intelligence (AI)-based solutions such as machine learning (ML) models have been employed in treating COVID-19 cases. However, limited application of AI for COVID-19 in Africa has been reported in the literature. This study aimed to investigate the performance and interpretability of several ML algorithms, including deep multilayer perceptron (Deep MLP), support vector machine (SVM) and Extreme gradient boosting trees (XGBoost) for predicting COVID-19 mortality risk with an emphasis on the effect of cross-validation (CV) and principal component analysis (PCA) on the results. For this purpose, a dataset with 154 features from 490 COVID-19 patients admitted into the intensive care unit (ICU) of Tygerberg Hospital in Cape Town, South Africa, during the first wave of COVID-19 in 2020 was retrospectively analysed. Our results show that Deep MLP had the best overall performance (F1 = 0.92; area under the curve (AUC) = 0.94) when CV and the synthetic minority oversampling technique (SMOTE) were applied without PCA. By using the Shapley Additive exPlanations (SHAP) model to interpret the mortality risk predictions, we identified the Length of stay (LOS) in the hospital, LOS in the ICU, Time to ICU from admission, days discharged alive or death, D-dimer (blood clotting factor), and blood pH as the six most critical variables for mortality risk prediction. Also, Age at admission, Pf ratio (PaO2/FiO2 ratio), troponin T (TropT), ferritin, ventilation, C-reactive protein (CRP), and symptoms of acute respiratory distress syndrome (ARDS) were associated with the severity and fatality of COVID-19 cases. The study reveals how ML could assist medical practitioners in making informed decisions on handling critically ill COVID-19 patients with comorbidities. It also offers insight into the combined effect of CV, PCA, and SMOTE on the performance of ML models for COVID-19 mortality risk prediction, which has been little explored.

## Introduction

The effect of the coronavirus disease 2019 (COVID-19) pandemic on healthcare systems all over the world has been devastating^1^. As a result, various clinical intervention methods have been employed in the detection, diagnosis, and prognosis of COVID-19 cases, including clinical/laboratory methods and medical image-based diagnosis^2,3^. Lately, the use of artificial intelligence (AI) methods for tackling the challenges of COVID-19 has received significant attention. Some of the efforts so far reported include the application of machine learning (ML) as a subbranch of AI to detect COVID-19 infections^2,4,5^, the prognosis of COVID-19 progression^6–10^, and the determination of COVID-19 treatment outcomes^11–14^. However, limited instances of applying AI to the prognosis of COVID-19 cases in Africa have been reported in the literature.

South Africa recorded the highest number of COVID-19 cases in Africa (http://www.worldometers.info/coronavirus). A large percentage of the population also has pre-existing comorbidities such as human immunodeficiency virus (HIV), tuberculosis (TB), and diabetes. comorbidities in patients with COVID-19 complicate prevention strategies and are more difficult to manage

^15^. AI-enabled decision-making for treating COVID-19 can enhance healthcare quality, particularly in settings with a paucity of medical expertise^16^. However, this is not common in many African countries. Healthcare practitioners mostly rely on observations from clinical examinations of patients and their own experiences when making clinical decisions.

Recently, many instances of prediction and prognosis of COVID-19 using ML have been reported in the literature. ML algorithms have been shown to possess the ability to predict COVID-19 outcomes accurately. Specific areas of application of ML include the prediction of future situation of COVID-19 regarding emergence of new cases (infection), deaths within a country or across regions^4,5,13,17,18^; detection of COVID-19 infection, including prediction of COVID-19 outcome (death/survival) of critically ill patients^12,19–22^; the use of X-ray images^2,23–25^; and prediction of mortality risk and determination of the prognostic value of specific clinical variables^6–11,14^. Accurate COVID-19 mortality prediction is essential because it will enable health management systems to allocate adequate and appropriate healthcare resources to the most critical COVID-19 cases. However, most of the previous studies on COVID-19 mortality prediction adopted an approach that involves feature selection, model training, and comparison of the performance of different ML algorithms. Studies that investigate the effect of the selective application of treatments, such as cross-validation (CV), principal component analysis (PCA) based dimensionality reduction, and the synthetic minority oversampling technique (SMOTE), or their combinations, on the performance of ML models regarding COVID-19 mortality prediction are rare. Thus, an understanding of the effect of these treatments on the performance of ML models during predictive modelling of COVID-19 mortality is still lacking. This knowledge gap requires to investigate the effect of PCA, CV and SMOTE on the performance of ML models when they are used for COVID-19 mortality prediction.

CV assesses how much a trained ML model can generalise on an independent dataset^26^. It is a method designed to improve a predictive model's generalizability. PCA is a dimensionality reduction technique that enables large datasets to be transformed into one with reduced dimensions (fewer variables) that is easier to explore and can be processed more efficiently and faster by ML algorithms^26^. Generally, applying PCA can lead to accuracy trade-offs, but it could still be worthwhile in many cases. Hence, it is an option for ML researchers when dealing with large datasets. Also, we went further in this study to interpret the mortality risk predictions using the shapley additive exPlanations (SHAP) model to determine the most critical variables with prognostic value regarding COVID-19 mortality. This is to provide a basis for improved decision-making by medical experts.

Summarily, this study has two objectives. The first is to perform predictive modelling of the mortality risk of COVID-19 patients, which could guide decision-making on their treatment by experimenting with some specific treatments (CV, PCA, and SMOTE). The second is identifying the most critical variables determining mortality risk in critically ill COVID-19 patients.

The rest of this paper is organised as follows. Section "Methodology" presents the methodology of the study. Section "Results" provides the experimental results obtained from the three selected ML models when different options of CV and PCA were applied. Section "Discussion" discusses the results, while the paper concludes in Section "Conclusions" with a summary and plan for future work.

## Methodology

### Study design and setting

**Figure 1:**
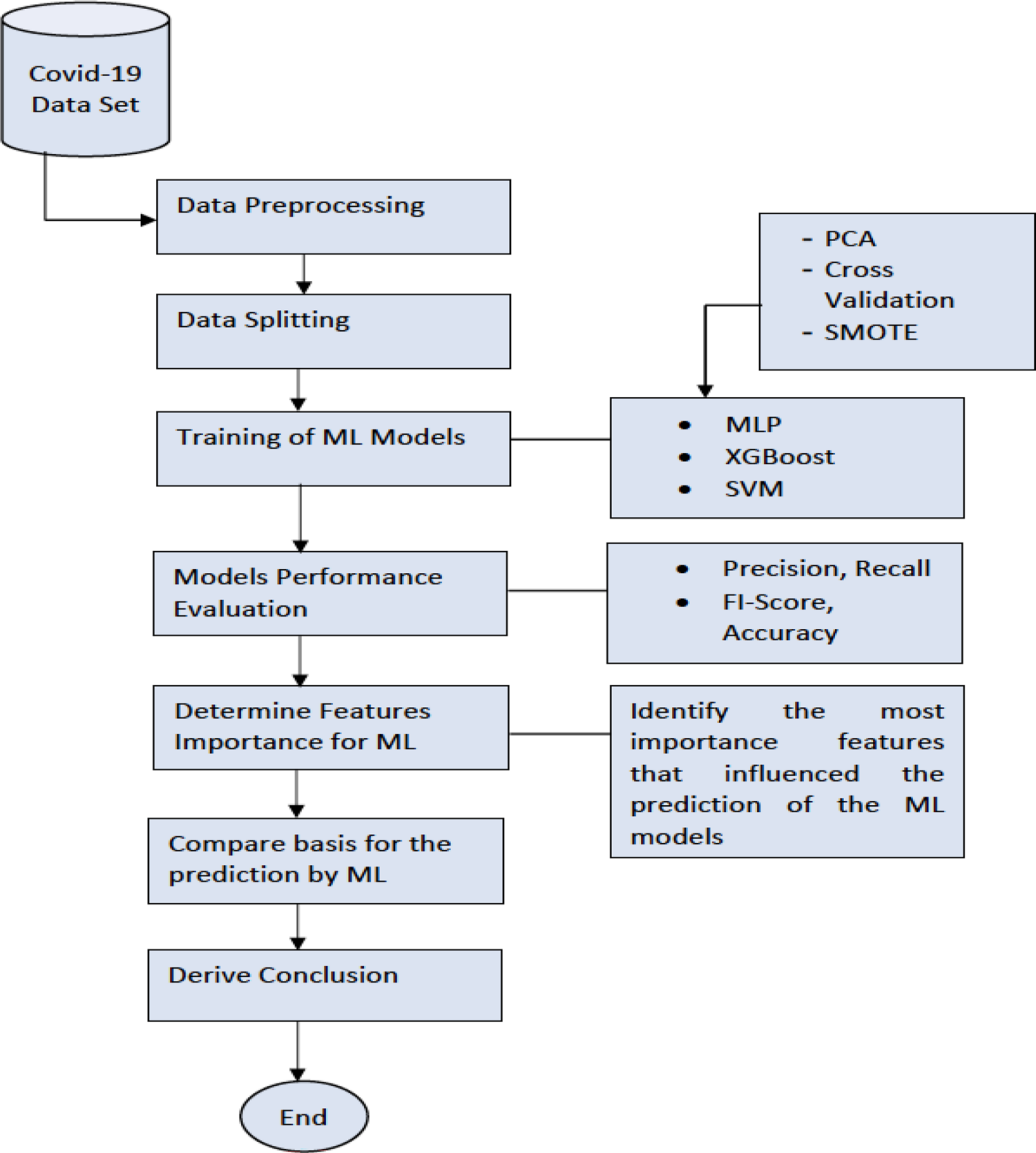
The workflow of the experimentation process.

The dataset used in this study pertains to a cohort of COVID-19 patients admitted into the ICU of Tygerberg Hospital in Cape Town, South Africa, during the first wave of COVID-19 from March to November 2020^27^. Figure 1 shows the process workflow of the experimentation that we did. Firstly, data was collected from the hospital. The dataset was then split into training and testing sets. The training set was used to train the Deep MLP, XGBoost and a SVM. Later, we evaluated the performance of the ML models on the test set using standard classification metrics. We benchmarked the performance of the models using the F1-score, accuracy, recall, precision and AUC score. The Classification Report method of Scikit Learn framework was used to generate the F1-score, accuracy, recall, and precision of the ML models. The report also contains the Macro Average (the average score across all classes for precision, recall, and F1) and the Weighted Average (the mean value of a metric per class (e.g., F1, recall, precision) while considering the support of each class).

After this, we applied the SHAP model, a unified framework for interpreting predictions by ML models to investigate the model interpretability^28^. SHAP is able to determine the level of the global importance of each feature to the prediction generated by a ML model. SHAP is a mathematical method based on game theory that can be used to explain any machine learning model's predictions by calculating each feature's contribution to the prediction^28^. We then compared the important features extracted through SHAP for each ML model to derive our conclusion.

### Predictor and outcome variables

The dataset consists of several aspects, such as:

- Socio-demographic and lifestyle characteristics (e.g. age, gender, ethnicity, residential area, occupation, workplace, smoking status, alcohol use, drug use etc.).
- Exposure history and symptoms (previous contact with infected persons or places; symptom types, severity score, etc.). This may not be available for critically ill patients.
- Comorbidities (such as Hypertension, chronic obstructive pulmonary disease (COPD), diabetes, heart diseases, TB, HIV, as well as the discharged alive/death outcome)
- Laboratory data such as full blood count (FBC), Ferritin, Procalcitonin (PCT), MicroRNAs, N-terminal pro–B-type natriuretic peptide (NT-proBNP) and inflammatory cytokine)
- Medication data (e.g., Steroids, antibiotics, antiviral, antifungal, medication for glycaemic control, antihypertensive etc.)
- Clinical monitoring – status (i.e., response to treatment) of the patient at the time of clinical examination, mechanical ventilation (e.g. Pulmonary Oxygen, Carbon Dioxide (CO2), Potassium, Calcium etc.)

The dataset consists of 154 features and 490 rows (records). There is one target variable–Discharged alive or Death. A snapshot of the dataset is presented in Tables 1 and 2. The dataset was imbalanced in terms of the target outcome (death or discharge) in the ratio of 60:35.

**Table 1:** Numeric/quantitative variables in the dataset.

s/no	Features	Range	Mean (STD)
1	Sodium	109–168	137.21 (5.38)
2	Potassium	3–8	4.48 (0.77)
3	Potassium +	2–7	3.9 (0.74)
4	Chloride	67–134	98.12 (6.16)
5	Standard bicarbonate	2–38	26.11 (4.9)
6	Hemoglobin A1C	1–22	8.12 (2.52)
7	Alanine transaminase	− 36–2948	65.14 (218.7)
8	Basophils	0–2	0.05 (0.22)
9	Creatinine	33–1807	99.37 (103.6)
10	D-dimer	0–18	3.84 (5.56)
11	Ferritin	− 456–44081	1709.53 (3409.16)
12	Haemoglobin	6–18	13 (1.85)
13	Hematocrit	0–1	0.022 (0.15)
14	International normalized ratio	1–5	1.05 (0.33)
15	Lymphocytes	0–54	2.61 (4.47)
16	Mean corpuscular haemoglobin concentration	26–36	32.03 (1.85)
17	Mean corpuscular volume	54–113	88.69 (6.74)
18	Monocytes	0–19	1.14 (2.05)
19	Mean platelet volume	7–15	9.71 (1.06)
20	Platelet count	56–967	310.32 (122.5)
21	Renal cell carcinoma	2–7	4.65 (0.75)
22	Urea	1–42	8.02 (5.64)
23	Red cell distribution width	11–21	13.72 (1.39)
24	Troponin T	2–1592	46.62 (124.1)
25	Procalcitonin	0–1806	6.19 (82.19)
26	White cell count	3–155	12.26 (8.18)
27	Total bilirubin	2–56	8.41 (5.03)
28	N-terminal pro–B-type natriuretic peptide	− 358–35000	1579.2 (3650.35)
29	Neutrophils	1–98	22.28 (27.89)
30	C-reactive protein	5–655	197.3 (114.3)
31	Fraction of inspired oxygen	21–100	75.22 (23.17)
32	PaO2/FiO2 ratio	11–871	94.91 (67.1)
33	Time to ICU from admission	0–21	1.46 (2.4)
34	Duration at ICU	0–61	7.71 (7.16)
35	Days discharged alive/death	0–61	7.71 (7.16)
36	Blood pH	6–8	7.23 (0.43)
37	Partial pressure of carbon dioxide	2–32	5.42 (2.39)
38	Partial pressure of oxygen in arterial blood	2–30	8.18 (3.69)
39	Lactate dehydrogenase	0–15	1.96 (1.64)
40	Oxygen saturation	2–182	86.66 (12.53)
41	Length of stay in hospital	0–81	11.63 (9.60)
42	Age at admission in complete years	16–81	52.9 (11.26)
43	COVID-19 Wave period	0–1	0.17 (0.37)
44	Weights	1–1	1 (0)
45	Fever	1–2	1.54 (0.50)
46	Cough	1–2	1.16 (0.37)
47	Sore throat	1–2	1.80 (0.4)
48	Rhinorrhoea	1–2	2 (0.09)
49	Myalgia	1–2	1.77 (0.42)
50	Fatigue	1–2	1.94 (0.23)
51	Headache	1–2	1.87 (0.34)
52	Joint pains	1–2	1.99 (0.09)
53	Dyspnoea	1–2	1.09 (0.28)
54	Nausea	1–2	1.98 (0.13)
55	Vomiting	1–2	1.97 (0.18)
56	Diarrhoea	1–3	1.90 (0.31)
57	Severe and critical condition	1–2	1.10 (0.30)
58	Oxygen Saturation	1–2	1.18 (0.39)
59	Arterial blood gas–Pa02	− 7–100	9.34 (12.32)
60	Temperature	33–100	41.33 (15.26)
61	Proteinuria	1–2	1.98 (0.15)
62	Haematuria	1–2	1.98 (0.13)
63	Acute physiology and chronic health evaluation II	3–9999	9823.37 (1184.6)
64	Pitt bacteraemia score	9999–9999	9999 (0.0)
65	Sequential organ failure	1–2	2 (0.06)
66	Symptoms of acute respiratory distress syndrome (ARDS)	1–2	1.74 (0.44)
67	Septic shock	1–2	1.98 (0.14)
68	Cardiogenic shock	1–2	2 (0.045)
69	Dysrhythmias	1–2	2 (0.064)
70	Symptom of Glasgow coma scale	− 1–99	16.26 (12.88)
71	Symptoms of acute kidney failure	1–2	1.89 (0.32)
72	Hypertension	0–2	1.59 (0.5)
73	Asthma	1–2	1.05 (0.21)
74	Diabetes mellitus	1–2	1.49 (0.50)
75	Insulin resistance	1–2	1.04 (0.2)
76	Ischaemic heart disease	1–2	1.02 (0.15)
77	Hyperlipidaemia	1–2	1.1 (0.3)
78	Body mass index	1–3	1.70 (0.47)
79	Post-transplant lymphoproliferative disorder	1–1	1 (0)
80	HIV status	1–2	1.11 (0.31)
81	Immunodeficiency	1–2	1.01 (0.078)
82	Active Pulmonary TB	1–3	2 (0.10)
83	Chronic lung disease	1–2	1.03 (0.16)
84	Chronic kidney disease	1–2	1.04 (0.19)
85	Anion gap	1–49	13.49 (4.97)
86	Anion gap without potassium	4–54	17.48 (4.93)

**Table 2:** Categorical/qualitative variables in the dataset.

S/no	Features	Value type	Frequencies
87	Date of birth	Date	
88	Blood pressure	High/normal/mild_hypertension/optimal/moderate_hypertension/severe_hypertension	191, 109, 109, 41, 36,4
89	Ventilation	Other/high flow/intubated/NIV/CPAP	206, 178, 89, 17
90	Gender	Male/female	251, 239
91	Antibiotics	Yes/no	303, 187
92	Antifungals	Yes/no	5, 485
93	Antivirals	Yes/no	86, 404
94	Anticoagulants	Yes/no	446,44
95	Corticosteroids	Yes/no	417,73
96	Check for other medications_select_1	Checked/unchecked	234, 256
97	Check for other medications_select_2	Checked/unchecked	325, 165
98	Used ventilator	Non-invasive/invasive	401, 89
99	Death	Death/discharged alive	326, 164

### Data pre-processing

The data pre-processing techniques were applied to clean up the data and transform the data to numerical form (the numeric data points were scaled [0–1] by using the min–max normalisation function, while categorical variables were encoded using one-hot encoding). The dataset contained some missing values; therefore, the multivariate imputation technique was applied to fix missing values. Features (variables) that had 30% or more missing values and those considered redundant due to duplication were removed. A total of 55 variables were removed from the dataset that was used for analytics. These consist of 44 variables with more than 30% missing values and 11 variables regarded as redundant. Thus, after pre-processing, we were left with 99 variables and 490 rows used for our experimentation. The numeric variables in the dataset are shown in Table 1, while the categorical variables are presented in Table 2.

### Principal component analysis (PCA)

PCA is a statistical process that converts the observations of correlated variables into a set of linearly uncorrelated variables with the help of orthogonal transformation, leading to a smaller version of the dataset with fewer variables considered significant(dimensionality reduction). These newly transformed (significant) features are called the Principal Components. Although reducing the number of variables in a dataset could impact the accuracy of results, the trade-off in easier visualisation of results and less computational complexity makes the training of machine learning models faster^29^. Using PCA is particularly useful when dealing with a dataset with high dimensionality where the number of columns is more than the number of rows of data. The process of PCA involves:

I. Ensuring that the ranges of continuous initial variables are standardised
II. Identifying correlations by computing the covariance matrix
III. Identify the principal components by determining the eigenvectors and eigenvalues of the covariance matrix
IV. Creating a feature vector to decide which principal components to keep, and
V. Recasting the data along the principal components’ axes.

### Cross-validation

Cross-validation enables the training of ML models by using different subsets of the training dataset and evaluating them using a unique portion of that training dataset per time to enhance the generalisation of a trained ML model. Some of the more known techniques of cross-validation include^30^:

I. *Leave One Out Cross-Validation (LOOCV)* This entails leaving out one data point from the dataset while the rest is used to train the ML model for each learning set. The process is repeated for each data point so that k samples will have k training sets and k test sets. The process can help to minimise bias since all data points are used.
II. *K-fold cross-validation* This entails dividing the training set into k-folds (k equal groups), where each group is called a fold. At any given time, k-1 folds are used to train the model while the reserved fold is used as the test set. The process is repeated over k iterations and ensures that a unique fold is used as the testing set for variant compositions of k-1 folds that are used for training. For example, assume that k = 10 on the first iteration, fold-2 to fold-10 are used for training, while fold-1 is used as the test set. On the second iteration, fold  − 1 + fold − 3 + fold − 4 + ‚Ä¶ fold-10 is used for training, while fold-2 is used as the test set. The same procedure of k-1 folds for training and a unique (reserved) fold for testing is repeated for all k (10) iterations.
III. *Stratified K-Fold Cross-validation* This method is a variation of the k-fold cross-validation designed for cases of imbalanced datasets. It involves selecting the value k and splitting the dataset into k-folds while ensuring that each fold contains approximately the same proportional representation of target classes as the complete data set. Then, selecting k-1 folds as the training set and the remaining fold as the test set;, and training the model with a particular k-1 folds training set per time. Thereafter, validating the model with the unique test set, and continuing the process for k iterations. In the end, obtaining the final average score of the model's performance.

### Models training

This section presents theoretical perspectives on the three MLP Deep MLP, XGBoost, and SVM used in this study.

To overcome above-mentioned challenges, three ML algorithms, including deep multilayer perceptron (Deep MLP), extreme gradient boosting trees (XGBoost), and support vector machines (SVM) were selected for our predictive analytics. Although no particular ML algorithm works best for every problem or dataset, the selected methods rank among the best classification algorithms when dealing with tabular datasets based on evidence from the literature and ML competitions like Kaggle^31,32^. For example, the multiple hidden layers in Deep ML allow it to model highly non-linear relationships in data. This is crucial for tasks where the relationship between inputs and outputs is complex and not easily captured by linear models^32,33^. SVM has good generalisation performance and error bounds in real-life application problems, while XGBoost supports k-fold CV during model training, helping to assess the model's performance and reduce the risk of overfitting^32^. The selected algorithms more detailed as follow:

#### XGBoost

Boosting is an ensemble technique that combines a set of weak learners and aggregates their predictions to deliver improved prediction accuracy. Generally, gradient boosting represents an ensemble of machine learning models composed of decision trees to solve classification and prediction tasks. The Extreme Gradient Boosted Trees (XGBoost) algorithm works by growing a set of classification and regression trees (CART) sequentially to ensure the misclassification rate is reduced during subsequent iterations^32,33^. Compared to general gradient boosting, XGBoost can prevent model over-fitting by incorporating two other features apart from the regularised loss function. Firstly, it uses the learning rate hyper-parameter to scale down the weights of each new tree to ensure that a single tree does not dominate leading to the final score. Also, it uses column sampling by using only column-wise data from the training data set to build each tree. Combining these three attributes makes XGBoost outperform traditional gradient boosting methods.

#### Deep MLP

The MLP is a model of an ANN. ANN emulates the human biological neural system to solve computational problems using adaptive learning. The nodes of an ANN called artificial neurons correspond to the human neuron. The MLP can be used to perform functional approximation to solve classification and regression problems.

Different variants of the backpropagation algorithm can be used to train the MLP network by exposing it to samples of input–output pairs. The difference between the network's output and the target output is fed back into the network to update the weights that connect the hidden-output layers and the input-hidden layers. The knowledge of the network is stored in the set of weights (w) that connect the neurons^34,35^. The computation at each neuron is the linear combination of all inputs and weights that feed into it. A Deep MLP is a feedforward ANN with multiple (more than one) hidden layers fully connected in a dense architecture. Various activation functions such as rectifier (ReLU), sigmoid, and hyperbolic activation functions can be used in the Deep MLP.

#### SVM

The SVM is a supervised learning model that can perform classification and regression tasks and is capable of handling both linear and non-linear problems. It establishes a margin to determine data points that fall into specific classes. A typical SVM operation entails finding the best hyperplane (separating line) that separates the classes in a dataset into distinct classes. The hyperplanes represent decision boundaries that determine the class to which specific data points are classified. The support vectors are the data points from different classes closest to a hyperplane. The distance between the support vectors and the hyperplane is called the margin. The SVM algorithm aims to find the optimal hyperplane (decision boundary) with the maximum (longest) margin to enable a maximum number of points to be correctly classified in the training set. According to^34^, the SVM is a function that correctly classifies two classes with a maximum margin. SVM works with two kernel parameters: the box constraint (c) and the curvature weight (Gamma). The c is a regularisation term set before training the model and used to control the penalty for misclassification. It is the trade-off between having a smooth decision boundary and being able to classify all data points in the training set correctly. The Gamma is a hyperparameter that determines the level of influence of a single training point.

#### Hyperparameters for training the ML models

The three ML models–DMLP, XGBoost, and SVM- were trained using optimal parameters generated by the grid search function in Scikit Learn. The optimal parameters that were automatically selected for training the models are shown in Table 3.

**Table 3:** Hyperparameters for training the ML models.

XGBoost: best parameters	MLP: best parameters	SVM: best parameters
Colsample_bytree = 0.75 Learning_rate = 0.3 Max_depth = 12 n_estimators = 500 n_jobs = −1 Subsample = 0.75	With PCA: Input layer units = 69 Hidden layer 1: units = 71 Hidden layer 2: units = 73 Output layer units = 1	Kernels = rbf Gamma = 0.01 C = 25
	Without PCA: Input layer units = 116 Hidden layer 1: units = 118 Hidden layer 2: units = 120 Output layer units = 1	
	Activation: Input layer (relu) Hidden layer 1 (relu) Hidden layer 2 (relu) Outer layer (sigmoid)	
	Loss = 'binary_crossentropy', Optimizer = 'RMSprop' Batch_size = 128 Epochs = 50 Fold = KFold(n_splits = 3)	

At first, we considered four options in our experimentation with the three ML models (Deep MLP, SVM, and XGBoost):

i. PCA + No cross-validation
ii. PCA + cross-validation
iii. No-PCA + No cross-validation
iv. No-PCA + cross-validation

After determining the two options that produced the best performance, we then applied the synthetic minority oversampling technique (SMOTE) to these two options to investigate the model performance for a balanced dataset. Thus, for the three ML models, we considered the options:

xxii. No-PCA + No cross-validation + SMOTE
xxiii. No-PCA + cross-validation + SMOTE

### Metrics for evaluation of the machine learning models

After training machine learning models, it is always essential to measure the performance of the trained model on the test data set. In this study, we used standard classification metrics such as precision, recall, F1-score, and the Area Under the Receiver Operating curve (AUC) score for each ML model selected for the study. These metrics are explained as follows:

i. *Precision* This is the measure of positive predictions made that are truly positive. It is the ratio of the number of true positives (TP) by the sum of true positives (TP) and false positives (FP). $Precision\left(P\right)=\frac{TP}{TP}+FP$
ii. *Recall* This measures how well the ML model can predict the true positive instances. It is the total number of true positives (TP) divided by the sum of TP and false negatives (FN). It is a measure of how well the most important occurrence is predicted. $Recall\left(R\right)=\frac{TP}{TP}+FN$
iii. *Accuracy* the percentage of prediction that is correct. It is measured by dividing the number of correct predictions by the total number of predictions. It is not a good measure to assess a ML model when the dataset is imbalanced^4^.
  $$Accuracy=Noofcorrectlyclassified\frac{samples}{total}numberofsamples$$
iv. *F1-score* The harmonic mean of precision and recall gives a more balanced description of model performance. It is a value between 0 and 1. The F1 score is a suitable metric for assessing model performance for an imbalanced dataset. $F-Measure=2\frac{PR}{P}+R$. According to^36^, FI score is interpreted as follows: F1 > 0.9 (very good); 0.8–0.9 (good); 0.5–0.8 (okay); < 0.5 (not good).
v. *AUC score* The Area Under the Curve (AUC) measures how well a classifier can distinguish between classes and is used as a summary of the receiver operating curve (ROC). The AUC score is rated as follows: excellent (0.9–1), good (0.8–0.9), fair (0.7–0.8), poor (0.6–0.7), and failed (0.5–0.6).

### Ethics approval and consent to participate

All methods used in this study followed relevant guidelines and regulations. Ethical approval and waiver of consent were obtained from the Health Research Ethics Committee of Stellenbosch University (Approval number: N20/04/002_COVID-19). The National Health Laboratory Service (NHLS) of South Africa also approved the use of their data for the study (Reference: SRSR3982441). The data analytics experimentation was approved by the Research Ethics Committee of the Faculty of Informatics and Design of the Cape Peninsula University of Technology (30/Daramola/2021).

## Results

The results obtained from evaluating the ML models when the six options were implemented are presented next.

### PCA without cross-validation

**Figure 2:**
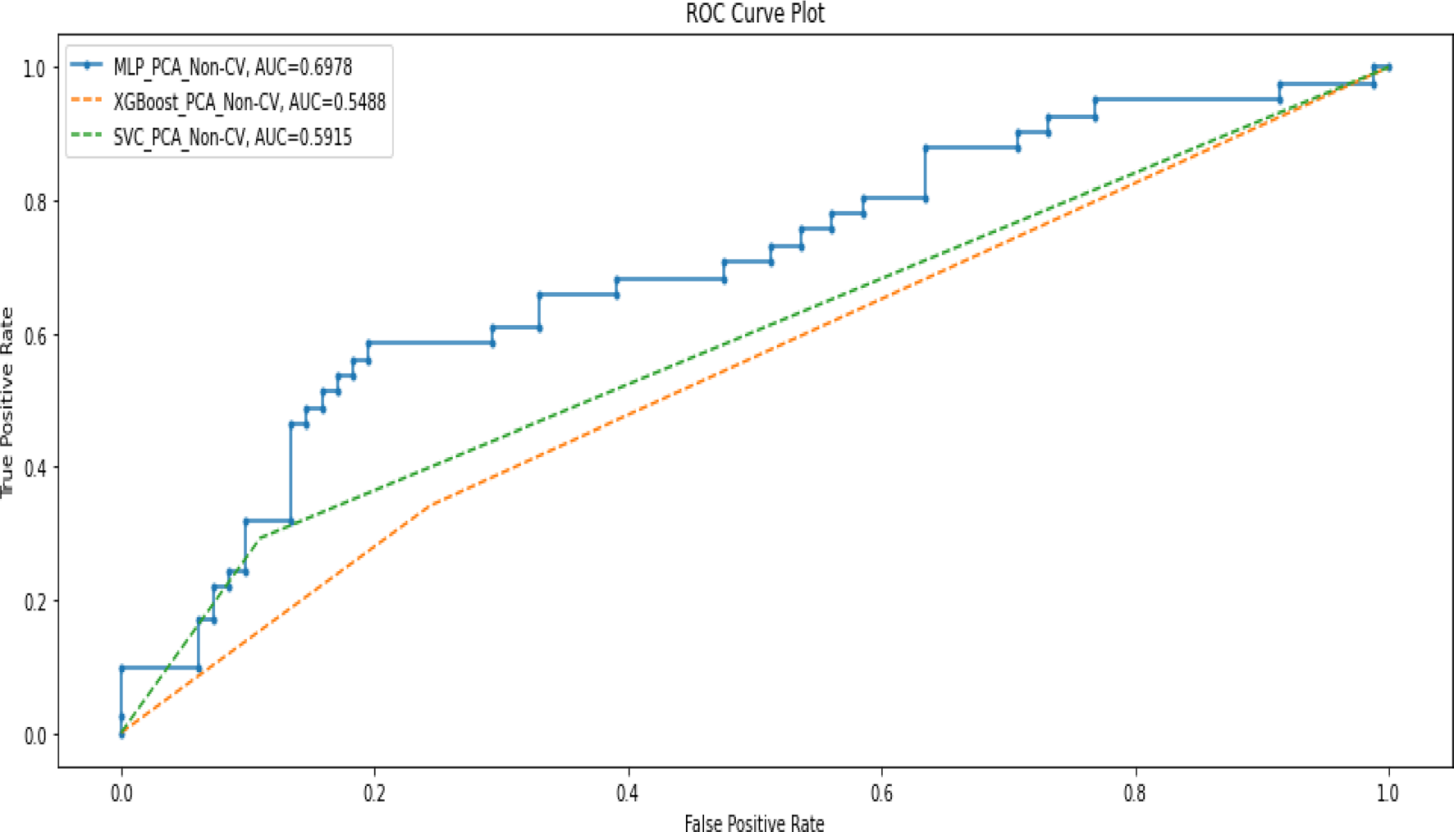
AUC Scores when models were trained with PCA data without cross-validation.

Table 4 shows the performance of the three models measured using precision, recall, F1-score, and accuracy when trained with data obtained after applying PCA on the original data without k-fold cross-validation. Figure 2 shows the AUC scores of the three models when trained under the same condition.

**Table 4:** Performance metrics when PCA was applied to training data without cross-validation.

Model		Precision	Recall	F1-score	Accuracy	AUC-score
Deep MLP	Death(0)	0.79	0.80	0.80	0.72	0.70
	Alive(1)	0.59	0.56	0.57		
	Macro average	0.69	0.68	0.69		
	Weighted average	0.72	0.72	0.72		
XGBoost	Death(0)	0.70	0.76	0.73	0.62	0.55
	Alive(1)	0.41	0.34	0.37		
	Macro average	0.55	0.55	0.55		
	Weighted average	0.60	0.62	0.61		
SVM	Death(0)	0.72	0.89	0.79	0.69	0.59
	Alive(1)	0.57	0.29	0.39		
	Macro average	0.64	0.59	0.59		
	Weighted average	0.67	0.69	0.66

### PCA with cross-validation

**Figure 3:**
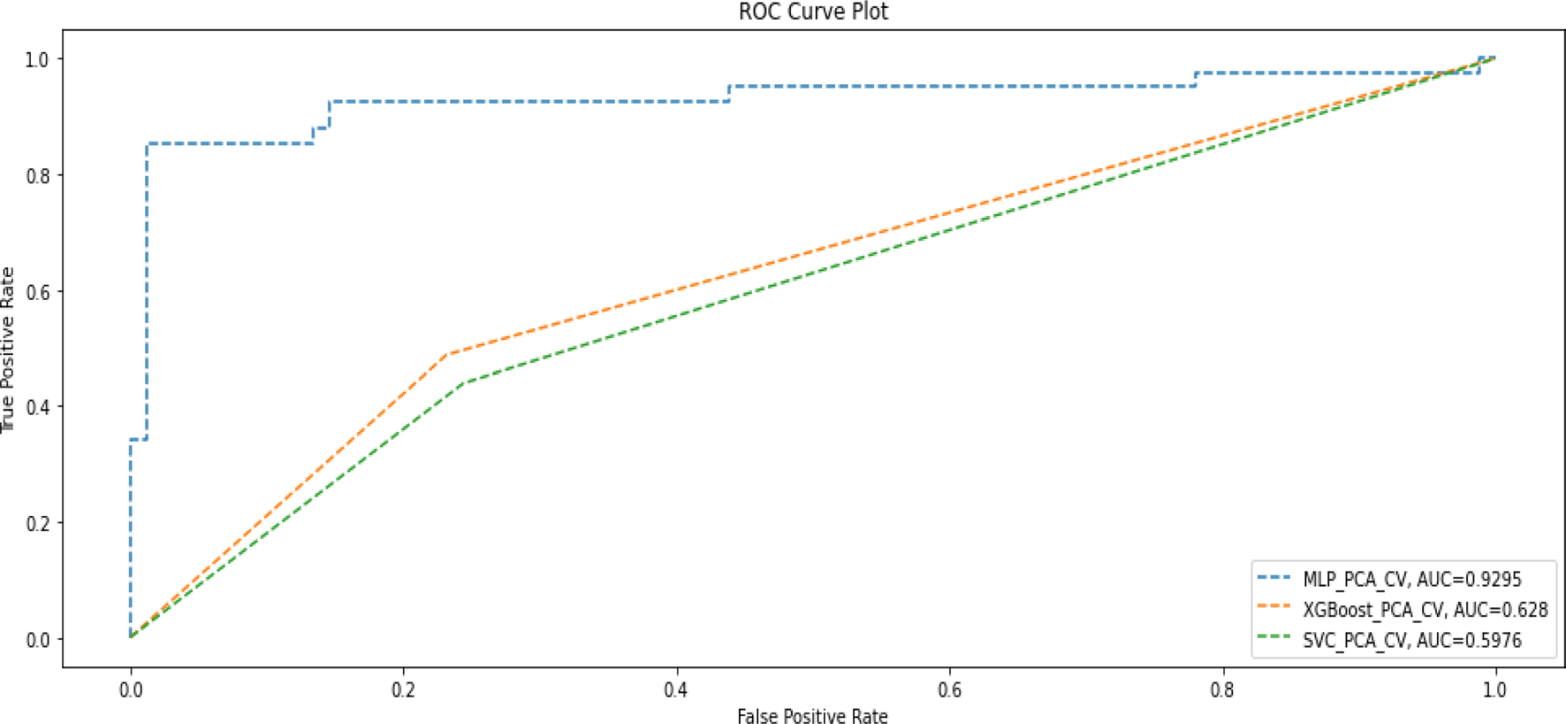
AUC Scores when both PCA and cross-validation were applied to training data.

Table 5 shows the performance of the three ML models after PCA plus k-fold cross-validation was applied to the training data, while Fig. 3 shows the AUC scores.

**Table 5:** Performance metrics when both PCA and cross-validation were applied to training data.

Model		Precision	Recall	F1-score	Accuracy	AUC-score
Deep MLP	Death(0)	0.92	0.95	0.94	0.91	0.93
	Alive(1)	0.91	0.84	0.87		
	Macro average	0.91	0.90	0.90		
	Weighted average	0.91	0.91	0.91		
XGBoost	Death(0)	0.75	0.77	0.76	0.67	0.63
	Alive(1)	0.51	0.49	0.50		
	Macro average	0.63	0.63	0.63		
	Weighted average	0.67	0.67	0.67		
SVM	Death(0)	0.73	0.76	0.74	0.65	0.60
	Alive(1)	0.47	0.44	0.46		
	Macro average	0.60	0.60	0.60		
	Weighted average	0.64	0.65	0.65

### Non-PCA without cross-validation

**Figure 4:**
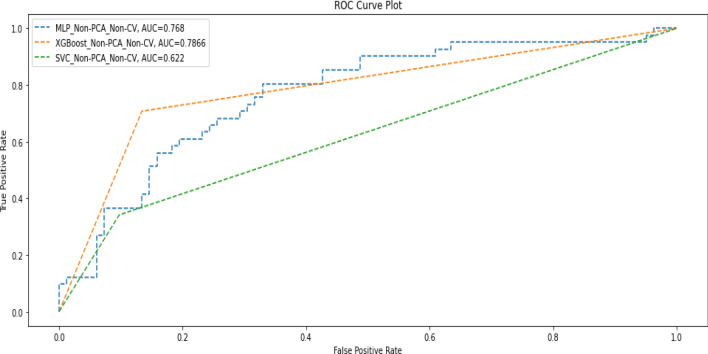
AUC Scores when none of PCA and cross-validation was applied for training.

Table 6 shows the performance of the three models when trained with the original data without using PCA or k-fold cross-validation, while Fig. 4 shows the AUC scores of the three models for the same scenario.

**Table 6:** Performance metrics when none of PCA and cross-validation was applied for training.

Model		Precision	Recall	F1-score	Accuracy	AUC-score
Deep MLP	Death(0)	0.80	0.79	0.80	0.73	0.77
	Alive(1)	0.60	0.61	0.60		
	Macro average	0.70	0.70	0.70		
	Weighted average	0.73	0.73	0.73		
XGBoost	Death(0)	0.86	0.87	0.86	0.81	0.79
	Alive(1)	0.72	0.71	0.72		
	Macro average	0.79	0.79	0.79		
	Weighted average	0.81	0.81	0.81		
SVM	Death(0)	0.73	0.90	0.81	0.72	0.62
	Alive(1)	0.64	0.34	0.44		
	Macro average	0.68	0.62	0.63		
	Weighted average	0.70	0.72	0.69

### Non-PCA with cross-validation

**Figure 5:**
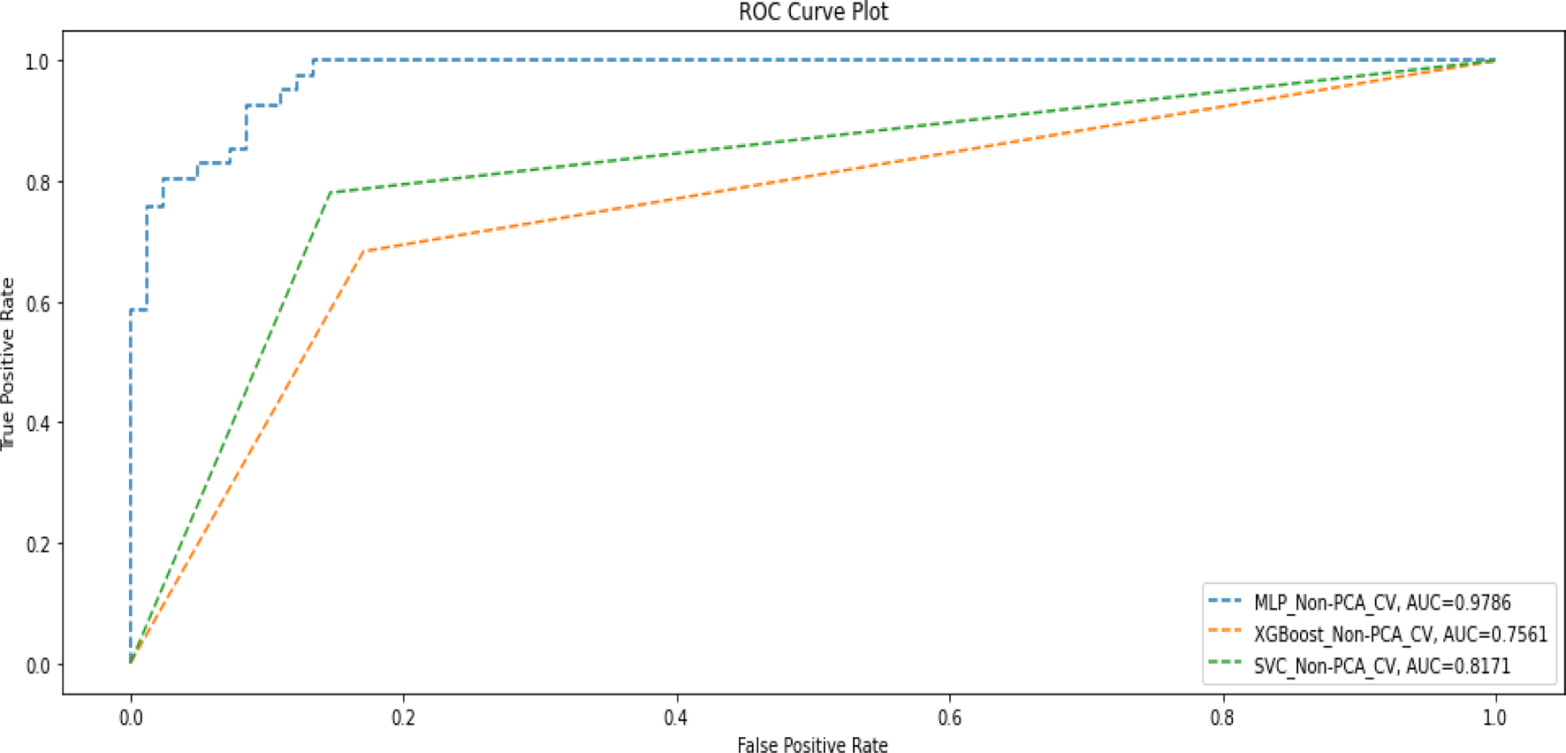
AUC score when models were trained without PCA but with cross-validation.

Table 7 shows the performance of the three models measured using precision, recall, F1-score, and accuracy when trained without applying PCA, but k-fold cross-validation was used. Figure 5 also shows the corresponding AUC scores of the three models.

**Table 7:** Performance metrics when ML models were trained without PCA but with cross-validation.

Model		Precision	Recall	F1-score	Accuracy	AUC-score
Deep MLP	Death(0)	0.80	0.86	0.83	0.76	0.98
	Alive(1)	0.67	0.56	0.61		
	Macro average	0.73	0.71	0.72		
	Weighted average	0.75	0.76	0.75		
XGBoost	Death(0)	0.84	0.83	0.83	0.78	0.76
	Alive(1)	0.67	0.68	0.67		
	Macro average	0.75	0.76	0.75		
	Weighted average	0.78	0.78	0.78		
SVM	Death(0)	0.89	0.85	0.87	0.83	0.82
	Alive(1)	0.73	0.78	0.75		
	Macro average	0.81	0.82	0.81		
	Weighted average	0.83	0.83	0.83

### Effect of applying the synthetic minority oversampling technique (SMOTE)

**Figure 6:**
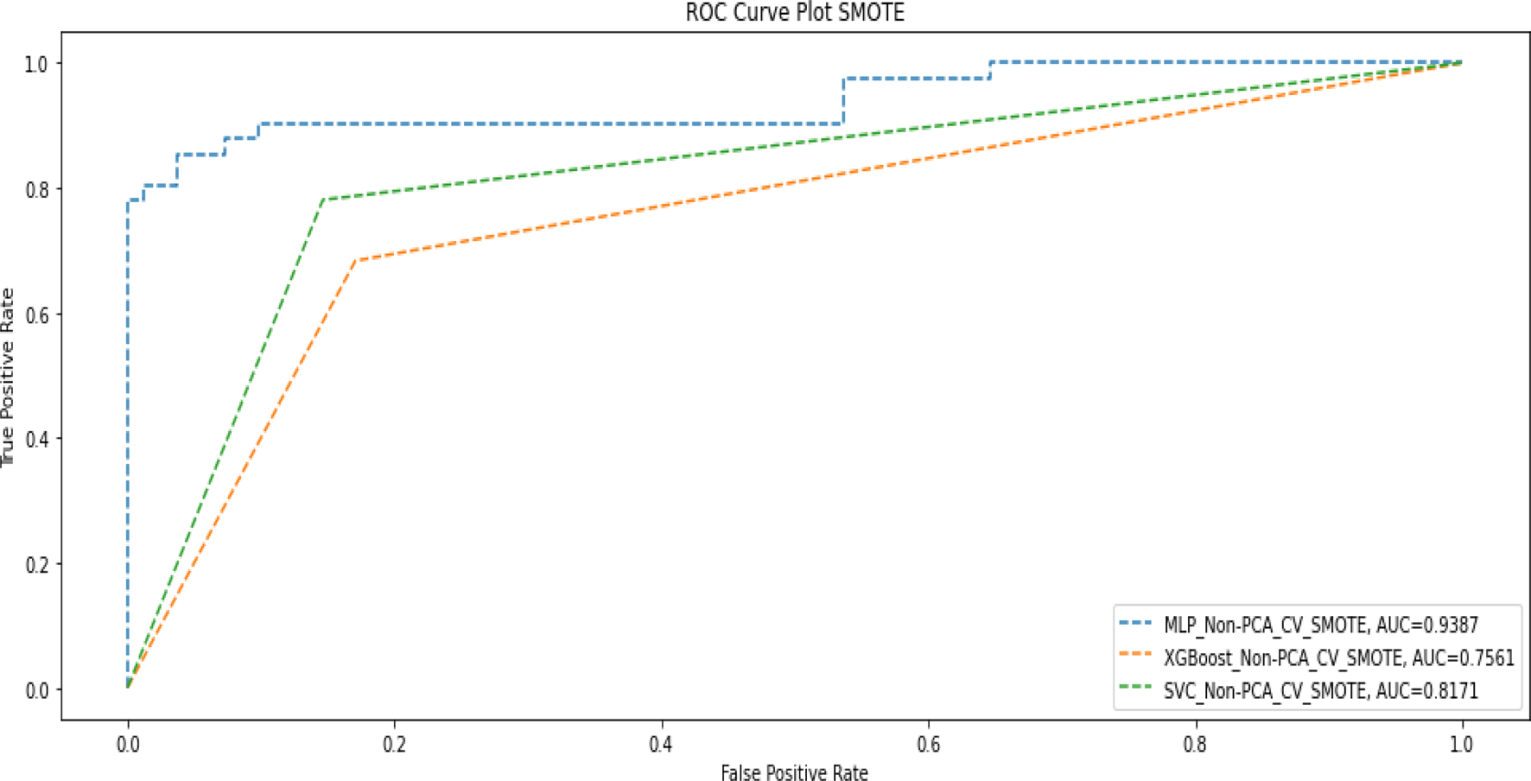
AUC score when SMOTE and cross-validation without PCA were applied.

**Figure 7:**
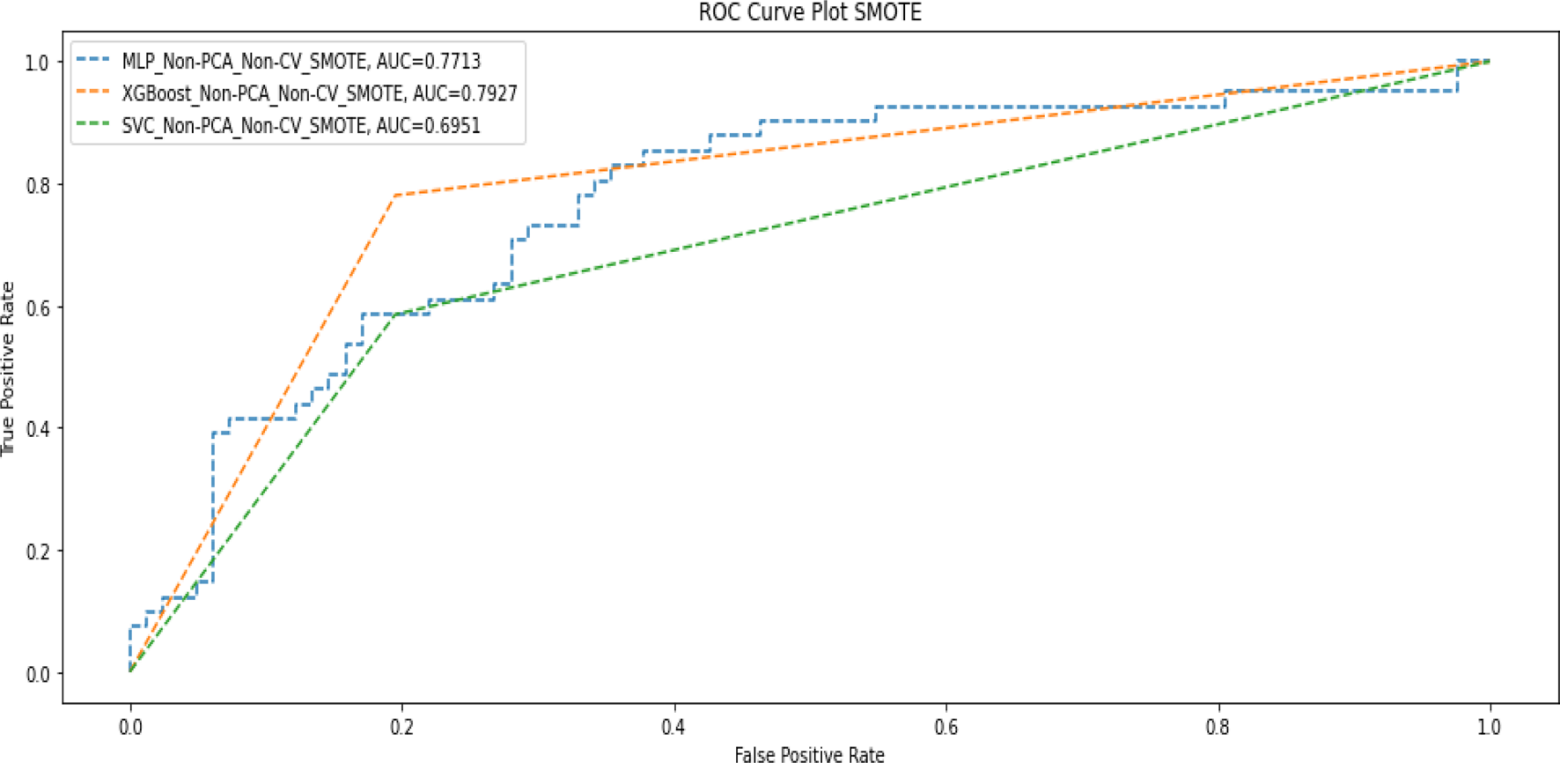
AUC Score when SMOTE, without cross-validation and PCA was applied.

The results obtained from our experiments on the initial four options showed that the ML models generally performed better when PCA was not applied. Since the dataset was imbalanced in terms of the target value in the ratio of 60:35, we applied SMOTE to increase instances of the minority class. This resulted in a training dataset of 488 records with an even distribution (50:50) of the target variable. We then compared instances of combining cross-validation plus SMOTE without PCA and when cross-validation and SMOTE without PCA were used. Tables 8 and 9 show the results obtained in these instances, while the corresponding AUC scores are shown in Figs. 6 and 7.

**Table 8:** Performance metrics when SMOTE and cross-validation without PCA were applied.

Model		Precision	Recall	F1-score	Accuracy	AUC-score
Deep MLP	Death(0)	0.91	0.98	0.95	0.93	0.94
	Alive(1)	0.96	0.81	0.88		
	Macro average	0.94	0.90	0.91		
	Weighted average	0.93	0.93	0.92		
XGBoost	Death(0)	0.84	0.83	0.83	0.78	0.76
	Alive(1)	0.67	0.68	0.67		
	Macro average	0.75	0.76	0.75		
	Weighted average	0.78	0.78	0.78		
SVM	Death(0)	0.89	0.85	0.87	0.73	0.82
	Alive(1)	0.73	0.78	0.75		
	Macro average	0.81	0.82	0.81		
	Weighted average	0.83	0.83	0.83

**Table 9:** Performance metrics when SMOTE, without cross-validation and PCA, were applied.

Model		Precision	Recall	F1-score	Accuracy	AUC-score
MLP	Death(0)	0.79	0.74	0.77	0.70	0.77
	Alive(1)	0.54	0.61	0.57		
	Macro average	0.67	0.68	0.67		
	Weighted average	0.71	0.70	0.70		
XGBoost	Death(0)	0.88	0.80	0.84	0.80	0.79
	Alive(1)	0.67	0.78	0.72		
	Macro average	0.77	0.79	0.78		
	Weighted average	0.81	0.80	0.80		
SVM	Death(0)	0.80	0.80	0.80	0.73	0.70
	Alive(1)	0.60	0.59	0.59		
	Macro average	0.70	0.70	0.70		
	Weighted average	0.73	0.73	0.73

The summary overview of the different experiments is shown in Table 10.

**Table 10:** Summary of Results after experiments.

	Deep MLP		XGBoost		SVM	
	F1	AUC	F1	AUC	F1	AUC
PCA + No-CV	0.72	0.70	0.61	0.55	0.66	0.59
PCA + CV	0.91	0.93	0.67	0.63	0.65	0.60
Non-PCA + CV	0.75	0.98	0.78	0.76	0.83	0.82
Non-PCA + No-CV	0.73	0.77	0.81	0.79	0.69	0.62
Non-PCA + CV + SMOTE	0.92	0.94	0.78	0.76	0.83	0.82
Non-PCA + No-CV + SMOTE	0.70	0.77	0.80	0.79	0.73	0.70

The results in Table 10 show that in terms of F1 score and AUC score, the Deep MLP had a generally good performance when cross-validation (CV) without PCA was applied but had the best overall performance when CV and SMOTE were applied without PCA (F1 = 0.92; AUC = 0.94). The SVM (F1 = 0.83; AUC = 0.82) had the second-best performance when CV and SMOTE were applied without PCA. Thus, we found that, generally, the performance of both SVM and Deep MLP can be enhanced through CV, and they also perform very well without PCA. The XGBoost model (F1 = 081; AUC = 0.79) had the best performance of the three models when we used the raw data without applying any of CV, PCA or SMOTE. XGBoost also performed well with SMOTE, but it is not affected by CV and performed worse in all cases when PCA was applied.

### Analysis of feature importance generated by ML models based on SHAP values

**Figure 8:**
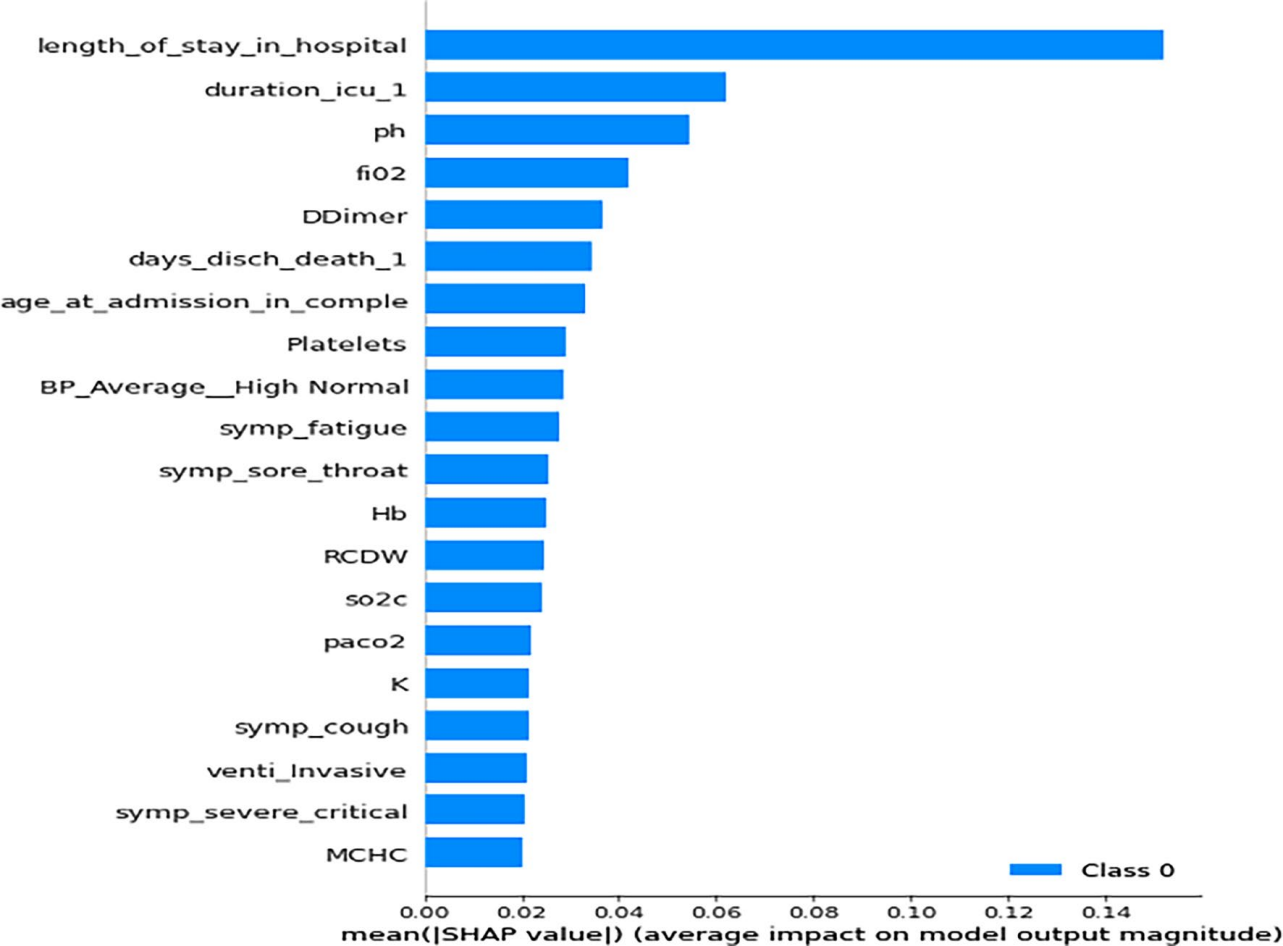
Important Features of the Deep MLP based on SHAP values.

**Figure 9:**
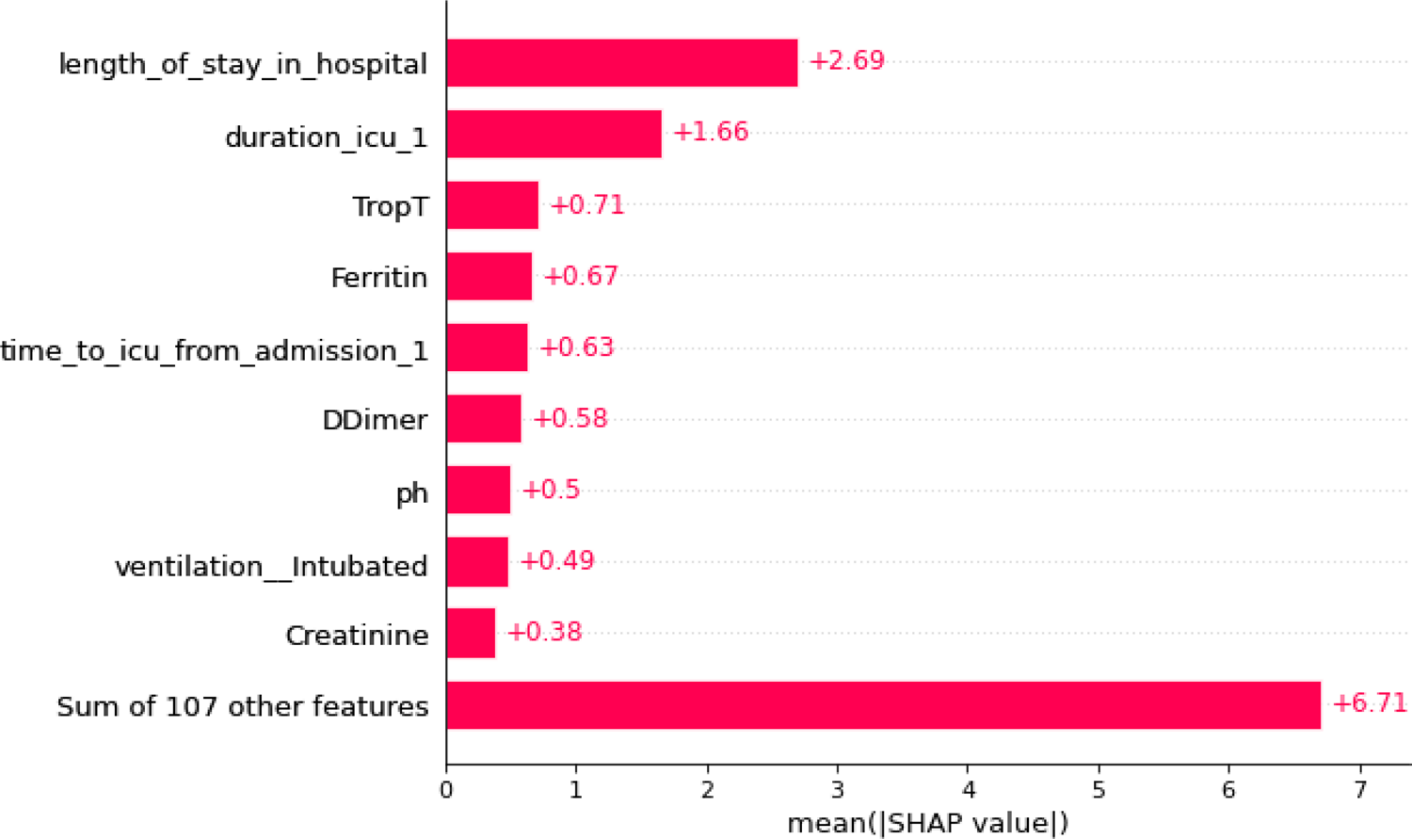
Important features to XGBoost based on SHAP values.

**Figure 10:**
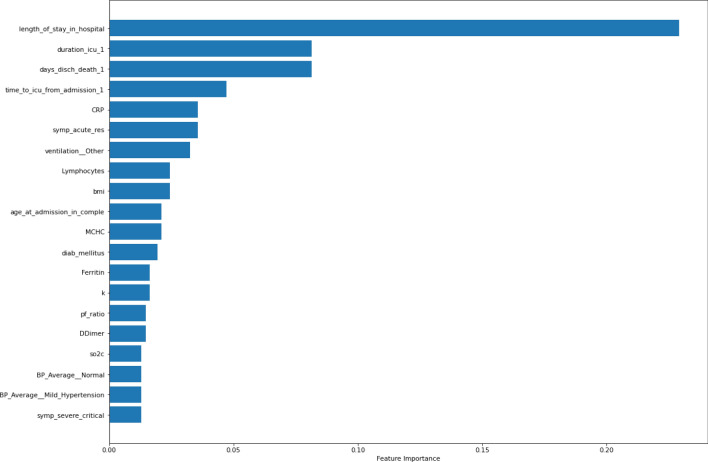
Important features of the SVM model based on SHAP values.

Based on the result of the experiments, we extracted instances where each of the three ML models had their best performance and examined the features that had the most influence on their prediction (feature importance) based on SHAP values. These are shown in Figs. 8, 9 and 10.

A comparison of the best-performing instances of the trained models is highlighted in Table 11, while the frequency of occurrence of specific features across the three ML models is shown in Table 12.

**Table 11:** Feature importance associated with predictions of ML models.

Non-PCA + Cross-validation		
MLP (F1 = 0.75; AUC = 0.98)	XGBoost (F1 = 0.78; AUC = 0.76)	SVM (F1 = 0.83; AUC = 0.82)
Length of stay in hospital Age at admission Duration in ICU Days discharged alive or death D-dimer pH FiO2	Length of stay in hospital Duration in ICU Trop Ferritin Time to ICU from admission D-dimer pH	Length of stay in hospital Days discharged alive or death Duration in ICU K Time to ICU from admission CRP Symp_acute_res

Non-PCA + No Cross-validation		
MLP (F1 = 0.73; AUC = 0.77)	XGBoost (F1 = 0.81; AUC = 0.79)	SVM (F1 = 0.69; AUC = 0.62)
Length of stay in hospital pH Days discharged alive or death Duration in ICU Age at admission D-dimer Symp_acute_res	Length of stay in hospital Duration in ICU Trop Ferritin Time to ICU from admission D-dimer pH	Length of stay in hospital D-dimer Days discharged alive or death Duration in ICU Time to ICU from admission Age at admission MPV

Non-PCA + Cross-validation + SMOTE		
MLP (F1 = 0.92; AUC = 0.94)	XGBoost (F1 = 0.78; AUC = 0.76)	SVM (F1 = 0.83; AUC = 0.82)
Length of stay in hospital Time to ICU from Admission pH Age at admission D-dimer Pf Ratio Days discharged alive or death	Length of stay in hospital Duration in ICU Ventilated_intubated Trop D-dimer pH Pf Ratio	Length of stay in hospital Duration in ICU Days discharged alive or death Time to ICU from admission CRP Symp_acute_res Ventilation

**Table 12:** Frequency of occurrence of important features by ML Models.

Feature	Deep MLP	XGBoost	SVM	Score
Length of stay in hospital	Yes	Yes	Yes	3
Time to ICU from Admission	Yes	No	Yes	2
Duration in ICU	No	Yes	Yes	2
Days discharged alive or death	Yes	No	Yes	2
D-dimer	Yes	Yes	No	2
pH	Yes	Yes	No	2
Age at admission	Yes	No	No	1
Pf Ratio	Yes	No	No	1
CRP	No	No	Yes	1
Symp_Acute_Res	No	No	Yes	1
Ventilation	No	No	Yes	1
Trop	No	Yes	No	1
Ferritin	No	Yes	No	1

From the results in Table 12, the six most critical variables for predicting the mortality or survival of COVID-19 patients were: *Length of stay in the hospital, Duration in ICU, Time to ICU from Admission, Days discharged alive or death, D-dimer, and Blood pH.* In addition, we also found that other features such as *Age at admission,* PaO2/FiO2 ratio (*Pf_ratio), TropT, Ferritin, ventilation, C-reactive protein (CRP),* and *Symptom of Acute respiratory distress syndrome (ARDS) (Symp_Acute_Res)* are also critical determinants of outcome in terms of survival or death of a patient.

## Discussion

We will discuss the results of our experiments from two perspectives that align with the goals of our study.

### Lessons from applying PCA and cross-validation

We noticed that applying PCA in combination with cross-validation has not been commonly used for predicting the mortality risk of COVID-19. Most previous approaches have focused on applying feature selection techniques, such as the Pearson correlation^37^, Chi-square^38^, and recursive feature elimination^22^, to identify the relevant predictors to use for prediction without considering applying PCA. Table 13 shows an overview of recent papers on mortality prediction of COVID-19. Our observation is that although our accuracy, F1-score, and AUC results compare favourably with those of previous studies, the instances where the effect of application of PCA + cross-validation + SMOTE are not yet prevalent. However, the results of our research and those of others, as shown in Table 13, emphasise the potency of machine learning as a viable tool to aid decision-making on handling of COVID-19 cases and other diseases whenever a quality dataset is available.

**Table 13:** Overview of previous studies on mortality risk prediction using ML Models.

Author	Dataset	Accuracy (%)	Precision (%)	F1-score (%)	AUC-score (%)
This study					
Deep MLP	Used 99 predictors with K-fold cross validation, PCA, and SMOTE as treatments. The dataset consists of 490 samples	93.0	93.0	92.0	94.0
SVM		73.0	83.0	83.0	82.0
XGB		78.0	84.0	78.0	76.0
Moulaei et al.^37^					
RF	Used 38 predictors, dyspnea, ICU admission, and oxygen therapy were found as the top three predictors. Smoking, alanine aminotransferase, and platelet count were found to be the three lowest predictors of COVID-19 mortality	95.03	94.23	–	99.02
XGB		94.25	92.43	–	98.18
KNN		89.56	80.11	–	96.78
MLP		91.25	87.19	–	96.49
LR		91.23	83.94	–	94.22
J48		92.17	89.97	–	92.19
NB		87.47	81.32	–	92.05
Aljameel et al.^8^					
LR	Furthermore, K-fold cross-validation was applied for data partitioning and SMOTE for alleviating the data imbalance. The dataset consists of 287 samples. Experiments were performed using twenty clinical features as predictors	86.3	–	88.0	91.0
RF		95.2	–	95.5	99.0
XGB		89.7		90.6	96.0
Pourhomayoun and M. Shakibi^11^					
ANN	Used 57 predictors for predicting COVID-19 mortality. K-fold cross-validation was applied during training	89.98	–	–	93.0
KNN		89.83	–	–	90.0
RF		87.93	–	–	94.0
SVM		89.02	–	–	88.0
LR		87.91	–	–	92.0
DT		86.87	–	–	93.0
Das et al.^20^					
LR	Used 45 predictors. The treatments applied were tenfold cross validation, SMOTE and Adaptive Synthetic (ADASYN) sampling approach	96.5	–	–	83.0
SVM		97.0	–	–	82.5
KNN		94.2	–	–	64.4
RF		97.2	–	–	78.0
GB		97.1	–	–	78.7
Zakariaee et al.^21^					
J48	Used 27 predictors including patient’s demographics, clinical features, history of personal diseases/comorbidity, laboratory results, CT-SS, and output variable. Chi-square was used for feature selection. K-fold cross validation and SMOTE were applied during training. The dataset had 815 records	91.7	86.7	92.2	93.9
NB		78.1	78	78.2	87.0
LR		80.7	80.2	80.8	88.9
MLP		94.8	91.7	94.9	97.0
SVM		81.2	80.1	81.5	81.2
K-NN		93.1	87.9	93.6	97.2
RF		97.2	94.8	97.3	99.9
XGB		95.6	92.0	95.8	98.2
Khadem et al.^22^					
RF (for DM)	Started with 40 features for predicting mortality of patients with COVID-19 and diabetes mellitus (DM), and those without diabetes mellitus Used the recursive feature elimination (RFE) technique based on three different classifiers (logistic regression, gradient boosting, and AdaBoost) to create three voter systems. The voter systems led to dropping of 20 features, so that 20 predictors were used for mortality prediction. The dataset consists of 477 records	82.0	–	–	80.0
RF (for Non DM)		80.0	–	–	84.0

*MLP* Multilayer perceptron; *RF* Random forest; *SVM* Support vector machines; *XGB* Extreme gradient boosting; *J48* A decision tree algorithm; *LR* Logistic regression; *NB* Naïve Bayes; *KNN* K-Nearest neighbour; *ANN* Artificial neural networks; *GB* Gradient boosting.

Based on the results of our study, we learnt the following about the three selected ML models:

i. The performance of deep MLP and SVM

Cross-validation can enhance the model performance of Deep MLP and SVM if the proper training parameters are selected. The Deep MLP had a generally good performance in terms of F1-score and AUC when cross-validation was applied (F1 = 0.92 (very good); AUC = 0.94 (excellent)). This observation was consistent irrespective of whether SMOTE was applied or not (see Table 10). The SVM also showed the same pattern because it had a good F1-score (0.83) and a good AUC score (0.82) with or without SMOTE when cross-validation was applied. These observations align with the postulations in the literature that model training can be generally enhanced through cross-validation to prevent overfitting and improve model generalisation on unseen data^26,30^. Thus, cross-validation is recommended when using Deep MLP and SVM.

ii. The effect of PCA on the performance of ML models

We found a reduction in model performance when PCA was applied to the three ML models (Deep MLP, SVM, XGBoost). However, the Deep MLP performed well when PCA plus cross-validation was applied. Considering that we did not have a large data set, the drop in model performance when PCA was applied compared to when it was not was quite significant (see Table 9). This observation suggests that the choice of whether to apply PCA on a tabular dataset should be weighed carefully depending on the choice of ML model for predictive analytics. Based on our observation, PCA plus cross-validation could be a good choice when using deep learning models. Our findings in this study also support this notion. However, for the other ML models, there may be better results in accuracy. Our perspective on this, which is reinforced by the result of our experimentation, aligns with an established school of thought that the application of PCA can lead to loss of accuracy but has benefits in terms of easier model visualisation and faster training, which could be a desirable trade-off for model accuracy in certain types of applications^26,30^. For the medical domain, where model prediction accuracy is paramount, applying PCA on a tabular medical dataset may not be the first option. Instead, feature selection based on correlations between the independent variable and the target variable to work with a reduced number of variables, particularly for large data sets, is a better option to achieve better model accuracy. In all cases, the right choice that suits a specific dataset in terms of either applying PCA, feature selection through correlation, or using the whole feature set should be investigated through experimentation.

iii. Performance of XGBoost

XGBoost is one of the most powerful ML algorithms, particularly when dealing with tabular (non-image) data. It is acclaimed for being successful in several Kaggle competitions. Based on our experimentation, we found that XGBoost (F1 = 0.81 (good); AUC = 0.79 (fair)) performed best when used with the raw dataset without any PCA, cross-validation or SMOTE when compared with the other two ML models (Deep MLP, SVM). This observation aligns with the acknowledged characteristics of XGBoost as profiled in the literature^38,39^. According to^39^, XGBoost has inbuilt regularisation, which avoids data overfitting problems. It also has an internal function for cross-validation and is well-equipped to deal with missing values. These features make XGBoost capable of dealing with raw data without needing external treatments that other ML algorithms require. Our experiment shows that an attempt to apply cross-validation externally to XGBoost leads to lower performance. Also, because it is sparse-aware, SMOTE yielded no significant performance improvement, while PCA caused a reduced performance.

### Lessons from COVID-19 mortality prediction

From our study, we found that the six most critical variables for the prediction of mortality or survival of the COVID-19 patients admitted into the ICU (see Table 12) are (i) length of stay in the hospital; (ii) the Length of time spent in the ICU (Duration in ICU); (iii) Time to ICU from Admission; (iv) the number of days spent in the hospital before discharge or death; (v) the blood clotting factor (D-dimer), and (vi) the blood pH of the patient.

The first four variables collectively indicate the severity of the patient's illness level at the time of admission and its effect on the outcome. In comparison, the last two variables indicate the comorbidity condition of a patient. D-dimer, which is a measure of the blood clotting factor of a patient, could be indicative of the existence or absence of a comorbidity. Studies have shown that D-dimer has prognostic value in detecting severity and fatality associated with COVID-19 cases^40–43^. According to^40^, D-dimer can predict severe and fatal cases of COVID-19 with moderate accuracy. In^44^, it was reported that elevated D-dimers (> 2590 ng mL^−1^) and the absence of anticoagulant therapy could predict pulmonary embolism (PE) in hospitalised COVID-19 patients with severe infections.

Similarly^45^, established a relationship between blood pH and fatal outcomes in critically ill COVID-19 patients at an intensive care unit. This also confirms the prognostic value of blood pH for COVID-19, which our ML models also detected. In addition^45^, found that Blood pH value, mean arterial pressure, base excess, troponin, and procalcitonin were highly significant prognostic factors of in-hospital mortality.

Apart from D-dimers and Blood pH, other factors include a*ge at admission,* PaO2/FiO2 ratio (*Pf_Ratio*)*, TropT, Ferritin, ventilation, CRP,* and *Symptom of Acute respiratory distress syndrome (ARDS)* also have a strong association with the severity and fatality of COVID-19 cases. This observation is supported by studies that have shown that the most frequently reported biological anomalies in COVID-19 patients include elevations of inflammatory markers such as C-reactive protein, D-dimers, Ferritin and interleukin-6^45–47^. In addition, these findings also significantly complement the results of three studies that were previously conducted on the dataset of the same cohort in South Africa, as reported in^48–50^. In^48^, the authors found that raised neutrophil count and neutrophil/lymphocyte ratio (NLR) were associated with a worse outcome. At the same time, age, female sex, and D-dimer levels were independent risk factors associated with mortality risk. The results of^49^ found seventeen categorical variables were associated with mortality among patients admitted to the ICU. These are: age, intubation, HIV positive status, arterial blood gas results, lactate, PF ratio, urea, neutrophil count, C-reactive protein (CRP), Procalcitonin (PCT), D-Dimer, NT-proBNP, troponin T, HbA1c, magnesium, aspartate transaminase (AST), and alkaline phosphatase (ALP). Lastly, in^50^, the result showed that age (above 48 years), requiring intubation, HIV status, procalcitonin (PCT), Troponin T, Aspartate Aminotransferase (AST), and a low pH on admission were significant predictors of mortality.

Incidences of abnormal indicators of D-dimer, Blood pH, C-reactive protein (CRP), Ferritin, and TropT are usually associated with patients with comorbidities such as Diabetes, obesity, coronary artery disease, hypertension, atrial fibrillation, and chronic heart failure (CHF), cancer, previous angina, peripheral arterial disease, previous myocardial infarction (MI), chronic kidney disease (CKD), cerebrovascular disease, and chronic obstructive pulmonary disease (COPD), HIV and many more^51,52^. Thus, our findings have identified some prognostic factors that strongly relate to comorbidities and the prediction of severity and outcomes of critically ill COVID-19 patients.

### Study limitations

This study used a dataset of critically ill patients under intensive care in a public South African hospital. A limitation of this study was the limited size of the dataset. It was occasioned by the fact that South Africa had the highest number of intensive care admissions during the first wave of COVID-19 and, to a lesser extent, during the second wave, when vaccines were not available, and there was generally limited knowledge of COVID-19 preventive care strategies. After the first and second waves of COVID-19, ICU admissions in most hospitals dwindled nationwide (South Africa), which resulted in reduced access to data on intensive care patients. For this reason, the findings of this study have limited generalizability in that the performance of these ML models may vary when applied to different datasets or healthcare settings. Still, we consider our study’s findings profound and valuable for scholarship because they align with the findings from several clinical studies on the prognosis of COVID-19 mortality^40–43^. Also, very few case studies pertaining to COVID-19 patients under intensive care from the African context have been reported in the literature.

## Conclusions

In this study, we investigated mortality risk prediction using a dataset of COVID-19 patients admitted into a South African hospital's ICU during the first wave of COVID-19. We found that the six most critical variables associated with predicting the severity of COVID-19 in the patients were Length of stay in the hospital, Duration in ICU, Time to ICU from Admission, Days discharged alive or dead, D-dimer, and Blood pH. In addition, we also found that other features such as Age at admission, PaO2/FiO2 (the ratio of arterial oxygen partial pressure (PaO2 in mmHg) to fractional inspired oxygen (FiO2)), TropT, Ferritin, ventilation, C-reactive protein (CRP), and Symptom of Acute respiratory distress syndrome (ARDS). To do this, we compared the performance of three machine learning models, namely Extreme Gradient Boosting Trees (XGBoost), Support Vector Machines (SVM) and Deep Multi-Layer Perceptron (Deep MLP) when specific treatments: PCA, cross-validation and SMOTE were applied during the model training.

This study makes a theoretical contribution by providing insight into the effect of the combination of CV, PCA, and SMOTE on the performance and interpretability of MLP, XGBoost, and SVM, which has not been previously explored to the best of our knowledge. Also, the study demonstrates how predictive modelling can be applied to identify variables with prognostic value for predicting the severity and outcome of critically ill COVID-19 patients from a study setting in South Africa, which is not prevalent in the literature.

This study has practical implications. Firstly, it reveals how machine learning could be used to identify the critical variables with prognostic value for predicting the severity and mortality of critically ill COVID-19 patients with comorbidities. It can aid healthcare service delivery in Africa, where medical decision-support mechanisms that can lead to increased productivity and efficiency of medical practice are not common. Secondly, the study offers an intellectual guide to help machine learning practitioners make informed decisions during popular ML models like Deep MLP, SVM, and XGBoost. It provides practical insights on how cross-validation, PCA, and SMOTE could be applied during model training and the likely expected outcomes.

Since AI relies on complex mathematics and statistics, it is essential to ensure that the model produces accurate and clinically relevant results. Decision-tracing practices should be developed based on the computations behind the model for user implementation. In future work, we will look into the creation of explicit inference rules and the development of a decision support tool (web app) that can guide medical practitioners when dealing with critical cases of COVID-19. Also, we will explore the adaptation of such an approach to other infectious diseases.

## Data Availability

The code implementation can be shared on request. Peter Nyasulu (nyasulu@sun.ac.za) can be contacted for the data.
